# Notes on Arab Horses1From Burckhardt’s Bedouins and Wahabys, printed in 1831.

**Published:** 1903-05

**Authors:** Franklin E. Hoskins

**Affiliations:** Beirout, Syria


### NOTES ON ARAB HORSES.[1]

Contributed by the Rev. Franklin E. Hoskins,
BEIROUT, SYRIA.

There are three breeds of horses in Syria: the true Arab breed, the Turkman, and the Kourdy, which is a mixture of the two former. The Arab horses are mostly small, in height seldom exceeding fourteen hands; but are ill formed and they all have certain characteristic beauties, which distinguish their breed from any other. The

[1] From Burckhardt's Bedouins and Wahabys, printed in 1831.

Bedouins count five noble breeds of horses, descended, as they say, from the five favorite mares of their prophet: Taueyse, Manakeye, Koheyl, Saklawye and Djulfe. These five principal races diverge into infinite ramifications. Every mare particularly swift and handsome, belonging to any of the five principal races, may give origin to a new breed, the descendants of which are called after her; so that the names of different Arab breeds in the desert are innumerable. On the birth of a colt of noble breed, it is usual to assemble some witnesses, and to write an account of the colt's distinctive marks, with the name of its sire and dam. These genealogical tables never ascend to the grandam, because it is understood that every Arab of the tribe knows by tradition the purity of the whole breed. Nor is it always necessary to have such genealogical certificates, for many horses and mares are of such illustrious descent that thousands might attest the purity of their blood.

The pedigree is often put into a small piece of leather, covered with waxed cloth and hung round the horse's neck. The following may serve as a specimen:

"GOD ENOCH. In the name of the most merciful God, the Lord of all creatures; peace and prayers be with our Lord Muhammad, and his family, and his followers, until the day of judgment; and peace be with all those who read this writing, and understand its meaning. The present deed relates to the greyish-brown colt, with four white feet, and a white mark on the forehead, of the true breed of Saklawye, called Obeyan, whose skin is as bright and unsullied as milk, resembling those horses of which the prophet said: 'True riches are a noble and fierce breed of horses;' and of which God said: 'The war horses, those which rushed on the enemy with full-blowing nostrils; those which plunge into battle early in the morning.' And God spoke the truth in his incomparable book. This Saklawye colt was bought by Khoshrun, the son of Emheyt, of the tribe of Zebaa, an Anezeh Arab. The sire of this colt is the excellent bay horse called Merjan, of the breed of the Koheylan: its dam is the famous white Saklawye mare, known by the name of Jeroua. According to what we have seen, we attest here upon our hopes of felicity, and upon our girdles, O sheikhs of wisdom, and possessors of horses, that this grey colt above mentioned is more noble even than his sire and dam. And this we attest according to our best knowledge, by this valid and perfect deed. Thanks be to God, the Lord of all creatures!

"Written on the 16th of Safar, in the year 1223,

"Witnesses, etc."

The Arabs almost exclusively ride their mares and sell the horses to towns-people or farmers. While the price of a horse in Syria ranges from $40 to $500, the mares will average at least six times those amounts. Then the Arabs seldom consent to sell the whole of a mare. If he sells half, the buyer takes the mare, but is obliged to let the seller have the mare's next filly, or else to return the mare and keep the filly to himself. If the Arab has sold but one-third of his mare, the purchaser takes her home, but must give to the seller the fillies of two years, or else one of them and the mare. The fillies of the third year, and all subsequent, belong to the buyer as well as the male colts, whether produced in the first or any following year. This contract the Arabs call "selling one-half or one-third of the mare's belly," and thus it happens that most of the Arab mares are the joint property of two or three persons, or even half a dozen if the price of the mare be very high.

Immediately after the birth of a colt the Arabs tie its ears together over its head with a thread, that they may assume a fine pointing direction; at the same time they press the tail of the colt upwards, and take other measures whereby it may be carried high. The only care taken of the dam after she has produced her colt is to wrap a piece of cloth or linen round her body; this cloth is removed on the next day. If the mare is only partially possessed by an Arab, he is obliged, on the ninth day after she has produced a filly, to assemble some witnesses, and to declare before them his intention of giving the newborn filly to the seller of the mare, or to keep the filly and to return the mare to her former owner. Having once made this declaration, he is bound to abide by it. The colts remain with the mother thirty days, after which the Arabs always wean them, and then either give them to the seller of the mare or rear them themselves on camels' milk. For the space of 100 days after the colts have been weaned, it is not permitted to give them any other food than camels' milk; even water is not allowed. After that time the colt receives a daily portion of wheat diluted with water: at first only a handful; this quantity is gradually increased, but milk still continues to be the colt's principal food. Such is the colt's diet for 100 days more, during the latter of which he is allowed to feed upon the grass near the tents and drink water. The second period of 100 days having elapsed, barley is given to the colt; and if camels' milk be abundant in the owner's tent, a bucket of it is given every evening along with the allowance of barley.

It is well known that the Arabs are not so nice in the choice of a stallion as the European breeders; for they ascribe the good qualities of the colt to the dam rather than to the sire. I have heard, however, of Arabs who took their mares a journey of several days that they might breed by some celebrated horse; the price paid on such an occasion is usually one dollar or a sheep. The Arabs keep their horses in the open air all the year round; they never clean or rub them, but are careful in walking them gently whenever they return from a ride. From the time that a colt is first mounted (which is after its second year) the saddle is but seldom taken off its back; in winter time a sackcloth is thrown over the saddle; in summer the horse stands exposed to the midday sun.

The Arabs believe that some horses are predestined to evil accidents; and, like the Osmanlys, they think that the owners of other horses must sooner or later experience certain misfortunes, which are indicated by particular marks on the horses' bodies. Thus, if a mare has a star on the right side of the neck, they believe that she is destined to be killed by a lance. If the star be on one of the shank bones, the owner's wife will prove unfaithful to her husband, and the orthodoxy of the latter is liable to suspicion. There are above twenty evil marks of this kind, which have at all events the bad effect of depreciating the horse's value by two-thirds or more. The Arabs do not mark their horses, as some imagine; but the hot iron, which they frequently apply in curing a disease, leaves an impression on the skin that appears like an intended mark.

---

An Auspicious Alliance. A significant sign of the times, and one that meets with our heartiest approval, is the recent election of S. B. Nelson, D.V.S., Professor of Veterinary Medicine and Comparative Pathology in the Washington State Agricultural College, to the presidency of the State Board of Health. Veterinary medicine is becoming our most valuable and valued ally in the fight against disease, and Dr. Nelson's election is not only a graceful recognition of this fact, but also a deserved tribute to his long and faithful service as Secretary of the Board. We are proud of the rapid progress made of late years by our sister profession, and glad to recognize such men as Professor Nelson, of Washington, and Dr. E. N. Hutchinson, of Oregon, as our professional brethren.—*Medical Sentinel*, February, 1903.

The Journal is in receipt of Division of Veterinary Science New Zealand Department of Agriculture, Report 1901–'02, J. A. Gilruth, M. R. C. V. S., Principal Veterinary Officer and Bacteriologist, Chief of Division.

